# Sub‐Microliter ^1^H Magnetic Resonance Spectroscopy for In Vivo High‐Spatial Resolution Metabolite Quantification in the Mouse Brain

**DOI:** 10.1111/jnc.16303

**Published:** 2025-01-18

**Authors:** Alireza Abaei, Dinesh K. Deelchand, Jan Kassubek, Francescois Roselli, Volker Rasche

**Affiliations:** ^1^ Core Facility Small Animal MRI Ulm University Ulm Germany; ^2^ Center for Magnetic Resonance Research University of Minnesota Minneapolis Minnesota USA; ^3^ Department of Neurology Ulm University Ulm Germany; ^4^ German Center for Neurodegenerative Diseases (DZNE) Ulm Germany; ^5^ Department of Internal Medicine II Ulm University Medical Center Ulm Germany

**Keywords:** cortical areas, high spatial resolution, magnetic resonance spectroscopy, metabolite profile

## Abstract

Proton magnetic resonance spectroscopy (MRS) offers a non‐invasive, repeatable, and reproducible method for in vivo metabolite profiling of the brain and other tissues. However, metabolite fingerprinting by MRS requires high signal‐to‐noise ratios for accurate metabolite quantification, which has traditionally been limited to large volumes of interest, compromising spatial fidelity. In this study, we introduce a new optimized pipeline that combines LASER MRS acquisition at 11.7 T with a cryogenic coil and advanced offline pre‐ and post‐processing. This approach achieves a signal‐to‐noise ratio sufficient to reliably quantify 19 distinct metabolites in a volume as small as 0.7 μL within the mouse brain. The resulting high spatial resolution and spectral quality enable the identification of distinct metabolite fingerprints in small, specific regions, as demonstrated by characteristic differences in *N*‐acetylaspartate, glutamate, taurine, and myo‐inositol between the motor and somatosensory cortices. We demonstrated a decline in taurine and glutamate in the primary motor cortex between 5 and 11 months of age, against the stability of other metabolites. Further exploitation to cortical layer‐specific metabolite fingerprinting of layer I–III to layer VI–V in the primary motor cortex, with the latter showing reduced taurine and phosphoethanolamine levels, demonstrates the potential of this pipeline for detailed in vivo metabolite fingerprinting of cortical areas and subareas.

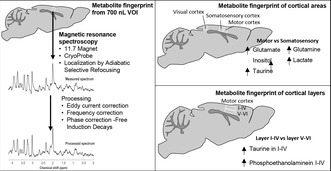

## Graphical abstract

An optimized acquisition and post‐processing pipeline for brain magnetic resonance spectroscopy based on the LASER sequence is reported, enabling metabolite spectra from 700 nL volume of interest in the murine brain in vivo. This pipeline is employed to demonstrate distinct neurochemical profiles in spatially close cortical areas (increased glutamate and lactate in motor cortex vs. somatosensory cortex) and the distinct neurochemical profile of different layers of motor cortex (increased taurine and phosphoethanolamine in upper layers). The new pipeline allows future studies characterizing the neurochemical and neurometabolic profiles of individual, identified cortical areas and sub‐structures.

**Abbreviations**
^1^H‐MRS: proton magnetic resonance spectroscopy
AFP: Adiabatic Full‐passage Pulses
Ala: alanine
Asc: ascorbate
Asp: aspartate
CP: cryogenically‐cooled radio frequency probe
Cr: creatine
CRLB: Cramér–Rao lower bound
CSDE: chemical shift displacement error
CV: coefficient of variation
ECC: eddy current correction
FID: free induction decays
GABA: γ‐aminobutyric acid
Glc: glucose
Gln: glutamine
Glu: glutamate
GPC: glycerophosphorylcholine
GSH: glutathione
HLSVD: Hankel Lanczos singular value decomposition
Ins: *myo*‐Inositol
Lac: lactate
LASER: localization by adiabatic selective refocusing
MRI: magnetic resonance imaging
NAA: N‐Acetylaspartate
NAAG: N‐Acetylaspartylglutamate
PCho: phosphorylcholine
PCr: phosphocreatine
PE: phosphorylethanolamine
PRESS: point‐resolved spectroscopy
RF: radio frequency
RRID: research resource identifier
sIns: *scyllo*‐Inositol
SNR: signal‐to‐noise ratio
Tau: taurine
TR/TE: repetition time/echo time
VAPOR: pulses of variable power with optimized relaxation
VOI: volume‐of‐interest

## 1 Introduction

Proton magnetic resonance spectroscopy (^1^H‐MRS) provides non‐invasive access to the neurochemistry of the brain in vivo. MRS is actively employed not only in the study of normal human and animal physiology (Lind et al. 2020), but is also highly relevant for the study of the pathophysiology of neurodegenerative, neoplastic, inflammatory, and vascular brain disorders (Lai and Niddam 2020; Maul, Giegling, and Rujescu 2020; Sanvito, Castellano, and Falini 2021; Unrath, Ludolph, and Kassubek 2007). The spatial metabolite profiling capabilities of MRS are further compounded by the multiplexing of the medium‐scale metabolite data with the high‐resolution anatomic details provided by structural magnetic resonance imaging (MRI). The acquisition of in vivo metabolites data of the brain has shown growing interest due to potential multiplexing with ex vivo spatial transcriptomics (Yao et al. 2023; Zhang et al. 2023) and ex vivo metabolomics imaging (Unsihuay, Mesa Sanchez, and Laskin 2021). However, while ex vivo transcriptomics and metabolomics are employed at cellular (or subcellular) resolution, and structural MRI can be easily performed with double‐digit micrometer resolution, the characterization of the in vivo metabolome has so far been constrained by substantially lower spatial resolution, limiting the transfer of information between imaging modalities (Foxley et al. 2021). In contrast to transcriptional or proteomic profiles, metabolites undergo rapid changes due to post‐mortem processes, chemical fixation, oxidation, and diffusion. Therefore, in vivo quantification using MRS remains a crucial technique for assessing pristine metabolic fluxes.

Currently, ^1^H‐MRS enables medium‐scale metabolite profiling in terms of absolute quantification (i.e., in μmol/g) measured within a landscape covering approximately 20 metabolites, including energy sources (creatine/phosphocreatine, glutamine, and glucose), neurotransmitters (glutamate and γ‐aminobutyric acid), metabolites involved in phospholipid metabolism (phosphoethanolamine, glycerophosphorylcholine, and phosphorylcholine), and molecules with diverse cellular functions (inositol, ascorbate, taurine, and N‐acetylaspartate; their physiological roles of these molecules have been previously explored (Rae 2014; Rae et al. 2024)), within a specific volume‐of‐interest (VOI) in the brain. However, the spatial resolution of ^1^H‐MRS metabolite profiling is limited by the signal‐to‐noise ratio (SNR) of the measured spectra. This limitation is influenced by factors such as the signal intensity, which is determined by the number of protons in the molecule/VOI, the acquisition duration and required averaging, the sensitivity of the receiver coil, and the post‐acquisition workflow (i.e., pre‐processing, spectral analysis, and quantification).

While the duration of the MR acquisition is intrinsically limited by ethical and biological consideration due to the animal tolerance to prolonged anesthesia, attempts to reach sufficient SNR levels have employed large VOI, ranging between 5 and 30 μL (Duarte, Do, and Gruetter 2014; Güell‐Bosch et al. 2020; Muraleedharan et al. 2020; Reyes et al. 2020), to increase the number of protons in the VOI and hence the MR signal. However, VOIs of this size are far from the single‐cell or single‐area resolution and actually rarely encompass a single homogeneous structure of the brain, and often unintentionally include nearby white matter, cerebrospinal fluid from ventricles and intervening gray matter (Güell‐Bosch et al. 2020).

Alternatively, further optimization of the SNR may be achieved by reducing the noise for example by introduction of low‐noise cryogenic RF transceiver coils (Ratering et al. 2008; Baltes et al. 2009) and adoption of specific MRS sequences. Several proton localized MRS sequences exist for in vivo MRS, including stimulated‐echo acquisition mode (STEAM) (Frahm, Merboldt, and Hänicke 1969), point‐resolved spectroscopy (PRESS) (Bottomley 1987), spin‐echo full‐intensity acquired localized (SPECIAL) (Mlynárik et al. 2006) and localization by adiabatic selective refocusing (LASER) (Garwood and DelaBarre 2001). The LASER sequence provides a spin‐echo signal with sharp 3D MRS voxel localization due to the presence of three pairs of adiabatic full‐passage pulses (AFP). In addition, the chemical shift displacement error (CSDE) is minimized owing to the large bandwidth of the AFP pulses. An interesting feature of utilizing pairs of AFP is that the apparent transverse relaxation time (T_2_) of water and metabolites are prolonged in addition to suppressing *J*‐modulation of *J*‐coupled metabolites such as glutamate, glutamine and *myo*‐inositol (Deelchand, Henry, and Marjańska 2015). To fully exploit the advantages of the LASER sequence, the preprocessing pipeline has been updated to include eddy current correction (ECC), frequency correction, and phase correction of individual free induction decays (FID). These features result in better spatial localization and SNR and were therefore recommended as the pulse sequence to use in preclinical studies by the MRS consensus group (Lanz et al. 2021).

The aims of this study were to: (1) develop a method for acquiring high‐quality, high‐SNR spectroscopic data in sub‐microliter volumes (< 1 μL) in the mouse brain in vivo; (2) reliably quantify metabolite concentrations in these small VOIs; and (3) demonstrate the application of sub‐microliter VOIs in resolving spectra from closely apposed cortical areas at single or multiple timepoints or even cortical layers. For this purpose, we have implemented a pipeline for high‐spatial resolution and large metabolite range quantification by applying a ^1^H transceive cryogenically‐cooled RF probe (CP) at 11.7 T field strength, a custom‐designed head restrainer, and a fully‐adiabatic 3D LASER sequence with built‐in frequency navigator. In combination with advanced preprocessing to increase the SNR and quantification of the metabolite concentrations using LCModel, the quantification of 19 metabolites from a VOI as small as 0.7 μL in mouse brains is reported.

## 2 Materials and Methods

### 2.1 Experimental Animals

C57BL/N6 mice were obtained from Charles River Laboratories, Sulzfeld, Germany. The experimental animals used were female mice, received from the vendor at approximately 5 months of age. At the time of the first MRI acquisition, they were approximately 5 months old and 11 months old at the time of the second acquisition. For the acquisition of multi‐region VOIs, 11 mice were used (although inclusion criteria for the spectra that passed the quality control were equal or smaller than 11: Olfactory bulb *n* = 11, Motor cortex *n* = 10, Somatosensory cortex *n* = 11, Visual cortex *n* = 8); for the acquisition at two timepoints, *n* = 5 mice were used; and for the acquisition of upper and lower cortical spectra, *n* = 8 mice were used. The mice were housed in a specific pathogen‐free facility maintained at a constant temperature of 23°C with a relative humidity of 50% (±5%). They were kept on a 12‐h light/dark cycle with ad libitum access to food and water. All animal experiments in this study were approved by the regional veterinary authority (Regierungspräsidium Tübingen, Germany, license no. C/II.205/1) and conducted in accordance with German animal welfare laws, regulations for the care and use of laboratory animals, and the institutional guidelines of Ulm University.

### 2.2 Sub‐Microliter VOI Resolution in ^1^H‐MRS

We first proceeded to optimize the MR acquisition. Several studies have reported a supralinear increase in SNR with increasing magnetic field and showed the advantage of using multi‐channel coils over single‐element surface coil at high fields (Pohmann, Speck, and Scheffler 2016; Tkáč et al. 2009; Wright and Wald 1997). However, room‐temperature coils are susceptible to thermal noise, which may become dominant in preclinical studies (as the sample size is typically diminutive). To mitigate this issue, we employed a cryogenically cooled RF coil, specifically a ^1^H transceive cryogenic quadrature RF probe (CP), operating at approximately 23 K, with a preamplifier cooled to around 77 K at the base (Niendorf et al. 2015). We also compared the performance of STEAM and LASER sequences in our optimization process. While STEAM (TR/TE/TM = 5000/2.7/10 ms) provided comparable spectral patterns, the LASER sequence significantly improved the SNR, with detailed parameters for LASER provided in the subsequent section.

Finally, we established an optimized pre‐processing pipeline for the raw acquired MRS data (Near et al. 2021; Tkáć and Gruetter 2005). This process includes eliminating eddy current effects and correcting for scanner drift or small animal motion (Near et al. 2021).

### 2.3 Acquisition of MRS Data

Experiments were performed at a dedicated ultra‐high field 11.7 T small animal system (117/16 USR BioSpec, AVANCE III, ParaVision 6.01 [RRID:SCR_001964], Bruker BioSpin, Ettlingen, Germany; https://www.bruker.com/en/products‐and‐solutions/preclinical‐imaging/mri/biospec/biospec‐117‐16.html) equipped with a 9 cm inner diameter self‐shielded gradient coil insert (B‐GA09S HP) providing 750 mT/m maximal strength in 80 μs rise time. A ^1^H cryogenically cooled 2‐element quadrature transmit/receive coil (CryoProbe, Bruker Biospin MRI GmbH, Ettlingen, Germany) was employed for excitation and signal reception. After initiation of the anesthesia with 5% isoflurane [Piramal critical care, Netherlands; Cat. No. 66794001710] in medical air (0.1 L/min), the mice were placed in a prone position in the animal holder. A custom‐built head restrainer was used to properly immobilize the animal's head during measurements, ensuring stability and reproducibility of the positioning in the experimental setup. The Isoflurane anesthesia gas was administered via a facial mask and during scanning, the isoflurane ratio was adjusted between 1.25% and 1.5% to maintain the respiratory frequency at about 90 cycles per minute.

Volume‐of‐interests (VOIs) were planned based on T_1_‐weighted multi‐slice FLASH images (TR/TE = 193/5 ms, flip angle = 17.5°). B_0_ Field homogeneity (1st and 2nd order terms) was adjusted for the investigated regions using a Bruker standard field‐map‐based approach (MAPSHIM) (Kim et al. 2002). A short echo‐time LASER (Localization by Adiabatic SElective Refocusing) sequence (Garwood and DelaBarre 2001) (TR/TE: 5000/16.75 ms: 10 kHz acquisition spectral width, 4096 data points and 386 averages) consisting of a 0.35 ms asymmetric sinc‐pulse (8.4 kHz bandwidth) followed by 3 pairs of 1.3 ms slice‐selective AFP HS1 refocusing pulses (13.8 kHz bandwidth, chemical shift displacement error of 3.6%/ppm) was used. Water signal was suppressed using seven 15.4 ms Hermite pulses of variable power RF pulses (bandwidth = 350 Hz) with optimized relaxation delays (VAPOR) (Tkác et al. 1999). The optimized reference pulse gain was manually determined by selecting the highest amplitude from a series of unsuppressed water single scans acquired at varying reference power levels to achieve the highest possible SNR. The refocusing pulses were automatically adjusted relative to the reference pulse.

We employed our MRS protocol to investigate the metabolite fingerprints of distinct but closely located cortical areas. In fact, ^1^H‐MRS spectra of single, homogenous cortical areas are difficult to obtain so far because the volume of a cortical area (often < 1 mm (Maul, Giegling, and Rujescu 2020) with a complex geometry) is smaller than the commonly employed VOI (between 5 and 20 μL—(Güell‐Bosch et al. 2020; Weerasekera et al. 2020; Zhu et al. 2019), resulting in the averaging of multiple, nearby cortical areas). We used the 0.7 μL VOI enabled by our proposed pipeline to explore the metabolite differences of primary motor cortex and primary somatosensory cortex; these two cortical areas are closely located but have substantial molecular, cellular and functional differences; we also recorded the ^1^H‐MRS of primary visual cortex, a sensory cortical area distant from the motor and somatosensory areas, and of the olfactory bulb. Total acquisition time per VOI (384 averages) was 32 min and 10 s. To mitigate the influence of anesthesia on metabolite concentrations, the order of VOI acquisition was randomized using a random‐number generator. Prospective B_0_‐field drift compensation was accomplished by updating the RF carrier frequency by utilizing a low flip angle navigator module in each repetition time (TR) (Henry et al. 1999). An unsuppressed water signal was also acquired in the same VOI to serve as both an internal reference and for eddy current correction. The water concentration was then refined within the VOI by accounting for the predominantly gray matter composition of the mouse brain, which constitutes approximately 81% of the tissue, thereby ensuring accurate quantification.

### 2.4 Processing of MRS Data

All stored single‐shot data were frequency and phase‐corrected based on internal navigator data. The preprocessing pipeline was meticulously updated to ensure the accuracy and reliability of the spectral data by incorporating eddy current correction (ECC), frequency correction, and phase correction of individual free induction decays (FIDs) using the MRspa (Magnetic Resonance spectral processing and analysis) software package, available online (Deelchand 2018). Initially, individual FIDs were averaged in groups of 12 to enhance the SNR and reduce random noise artifacts. Following this initial averaging, B_0_ inhomogeneity correction was performed using cross‐correlation methods, aligning each averaged FID to a reference frequency to mitigate frequency drifts and ensure precise alignment of spectral peaks across all FIDs. Concurrently, phase correction was executed through cross‐correlation techniques to eliminate phase inconsistencies, thereby enhancing the overall spectral quality. After these corrections, 32 blocks of 12 averaged FIDs each were summed to generate the final spectrum, effectively improving the SNR and enabling more reliable detection and quantification of metabolites, especially those present at lower concentrations or with overlapping signals. Subsequently, eddy current distortions were addressed using the ECC algorithm with a zero‐phase adjustment parameter within the MRspa software, effectively mitigating baseline distortions and preserving the integrity of the spectral data. This comprehensive preprocessing approach—comprising initial FID averaging, B₀ inhomogeneity correction, phase correction, summation of multiple averaged blocks, and eddy current correction—was systematically implemented using the MRspa software package, thereby enhancing the reliability and reproducibility of the spectral data and supporting robust downstream analyses.

Absolute metabolite concentrations were derived with LCModel Version 6.3‐1C (Provencher 1993) using in‐house simulated basis spectra based on density‐matrix formalism. The actual RF shapes and interpulse delays were taken into account. Nineteen metabolite spectra were generated using previously reported *J*‐coupling constants and chemical shifts (Govindaraju, Young, and Maudsley 2000). These include alanine (Ala), ascorbate (Asc), aspartate (Asp), creatine (Cr), γ‐aminobutyric acid (GABA), glucose (Glc), glutamate (Glu), glutamine (Gln), glycerophosphorylcholine (GPC), glutathione (GSH), lactate (Lac), *myo*‐inositol (Ins), N‐acetylaspartate (NAA), N‐acetylaspartylglutamate (NAAG), phosphocreatine (PCr), phosphorylcholine (PCho), phosphorylethanolamine (PE), *scyllo*‐inositol (sIns), and taurine (Tau). Metabolite‐nulled spectrum was also incorporated into the basis set to represent macromolecular (MM) contributions. This was obtained by employing a single inversion‐recovery module inserted prior to the LASER sequence (total averages = 384; TR = 3.5 s; inversion time = 0.85 s; VOI = 11.4 μL; HS1R23 inversion pulse duration = 4.6 ms). Residual metabolite resonances of total creatine (3.93 ppm), taurine, and choline, which were observed in the metabolite‐nulled spectrum due to differences in their T_1_ relaxation times (Tkác et al. 1999; Cudalbu et al. 2021), were removed using the Hankel Lanczos singular value decomposition (HLSVD) routine in MATLAB. All spectra were fitted between 0.5 and 4.2 ppm without any apodization or zero‐filling (Provencher 2001).

The reported SNR was defined as the ratio of the maximum peak of the NAA singlet to the root‐mean‐square noise measured between −2 and −4 ppm. Only metabolite concentrations with a Cramér–Rao lower bound (CRLB) ≤ 50% in at least half of the spectra per brain region were included in the statistical analysis. When a high correlation (*r* < −0.5, based on the Fisher matrix) existed between two metabolites, their combined concentration was reported. The coefficient of variation (CV) of the metabolite concentrations was calculated to characterize intra‐individual variability and assess reproducibility.

For each VOI of each animal, the following inclusion criteria were applied: (i) a water peak FWHM ≤ 17 Hz for optimal field homogeneity, (ii) no substantial motion during acquisition to prevent artifacts, (iii) stable physiological parameters throughout the experiment, and (iv) accurate voxel placement within the target region to avoid contamination. Data from visual cortex VOI of 3 animals and from 1 motor cortex VOI were excluded due to broader water peaks observed in the spectra or motion artifact.

The transverse T_2_ relaxation time of water tissue in the primary motor cortex was determined at 16, 42 and 54 weeks of age. A series of LASER experiments without water suppression was acquired with a constant repetition time (12 s), four accumulations, and varying echo times of 20, 24, 28, 35, 50, 70, 100, 150, 200, 250, and 300 ms. To determine the apparent water T_2_, the water signals were fitted to a bi‐exponential function in MATLAB.

### 2.5 Power and Sample Size Calculation and Statistical Analysis

Sample size and power calculations were performed using the G*Power 3.2 software [RRID:SCR_013726]. For the a priori sample size calculation, we used previously published data (Tkáč et al. 2004), focusing on the glutamate peak due to its high concentration and biological importance as an excitatory neurotransmitter. For the comparisons of multiple cortical areas (Figure 5), we considered approximately 20 analytes and four groups, with *α* = 0.05 and power = 0.80. Based on cortical glutamate levels of 12.3 ± 0.7 (according to Tkáč et al. 2004), we assumed a minimum detectable difference of approximately 10%, corresponding to a Cohen's f of 1.2–1.3 for a two‐way ANOVA, which resulted in a sample size of *n* = 12 per group. For the post hoc comparison, using a Cohen's *d* of 1.7 and similar power (with α = 0.008 after Bonferroni correction for multiple comparisons), we obtained *n* = 10. We elected to use *n* = 11 as an intermediate value. For comparing the same animals at two ages (Figure 6), we used a repeated‐measures *t*‐test with glutamate as the index analyte. Based on *α* = 0.05 and power = 0.80, with a minimum effect size of 10% (Cohen's *d* = 1.7, correlation = 0.5), we obtained a sample size of *n* = 5 per group. For the comparison of upper and lower cortical layers, we anticipated a smaller effect size (due to the proximity of the VOIs and their location within the same cortical area) of approximately *d* = 1.0 (approximately a 5% difference). Based on a repeated‐measures *t*‐test, with *α* = 0.05 and power = 0.80, this resulted in *n* = 8. These values are broadly consistent with the group sizes previously reported (Tkáč et al. 2004). Because we performed the calculation a priori, we did not take into account missing values or datapoints to be excluded due to technical quality failures.

Statistical analyses were performed using GraphPad Prism 8 software [RRID:SCR_002798]. The Shapiro–Wilk test was first applied to verify normality, and the Grubbs test was used to identify outliers (*α* = 0.05). Statistical comparisons of analytes across multiple cortical regions (Figures 4 and 6) were performed using a two‐way ANOVA followed by Sidak's post hoc test. For paired sample comparisons (Figure 5), a mixed linear model was employed to properly account for missing values. All values are presented as mean ± standard deviation.

## 3 Results

### 3.1 Achieving Sub‐Microliter VOI Resolution in ^1^H‐MRS

**FIGURE 1:**
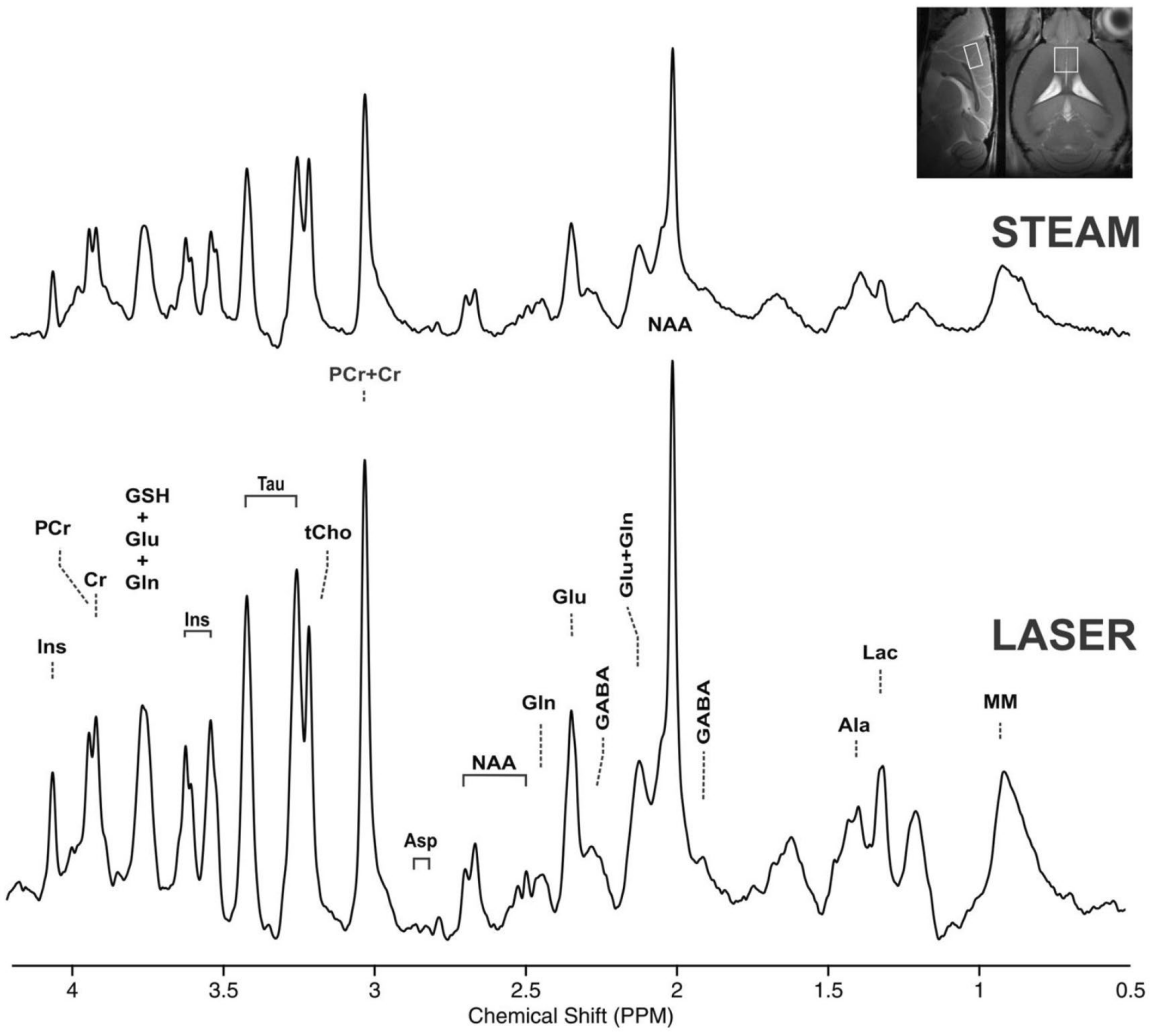
Comparison of STEAM (TR/TE/TM = 5000/2.7/10 ms, 256 averages, above) and LASER (TR/TE = 5000/16.74 ms, 256 averages, below) spectral quality acquired from a 7.1 μL volume of interest (VOI) located in the medial motor cortex of the mouse brain at 11.7 T. Data were acquired using a CP coil. Although the spectral patterns were comparable, the SNR, which reflects the signal intensity of metabolites, was substantially improved with LASER (138.8) compared to STEAM (71.4). All spectra were apodized with a 1 Hz line broadening and a Gaussian multiplication factor of 0.12 s for display purposes.

Figure 1 demonstrates that the spectral patterns obtained from ^1^H LASER and STEAM acquisitions in the medial frontal cortex of the mouse brain were comparable, despite the longer echo‐time associated with LASER. As anticipated, a higher SNR of the *N*‐acetylaspartate (NAA) peak at 2 ppm was achieved with LASER (138.8) than with STEAM (71.4) in the same animal.

**FIGURE 2:**
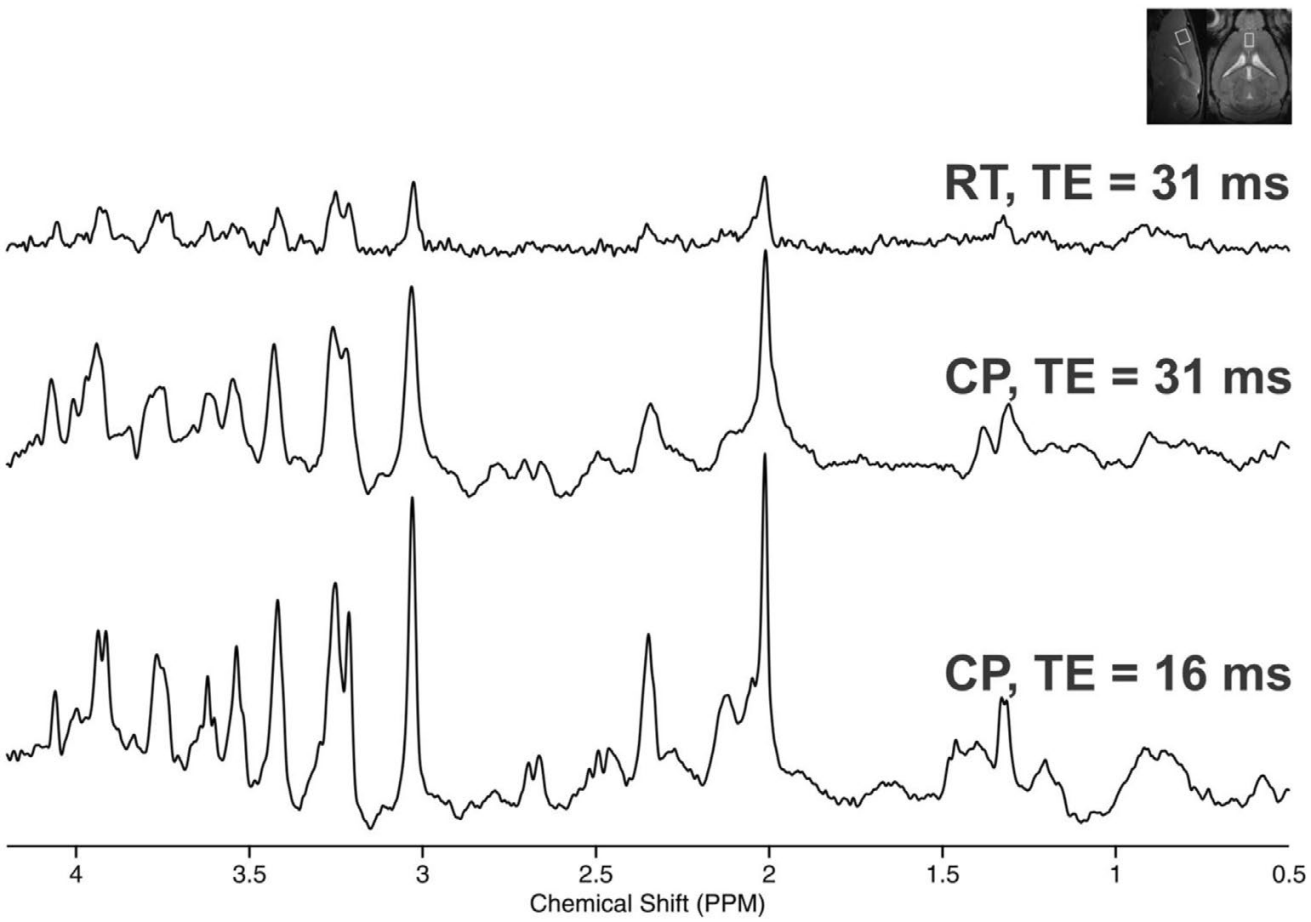
Comparison of spectral quality acquired using a room temperature (RT) coil and a cryocoil (CP). The RT coil consisted of a volume resonator for excitation and a four‐element phased‐array receive coil for signal reception. The minimum TE achieved with the RT coil was 31 ms. The CP coil consisted of two‐element transmit/receive coils, with a minimum TE of 16 ms. LASER MRS data (TR = 5000 ms, 256 averages) were acquired using the RT coil at a TE of 31 ms (minimum achievable TE), and using the CP coil at TEs of 16.74 ms (minimum achievable TE) and 31 ms for direct comparison with the RT coil. A VOI of 2.76 μL was positioned in the medial motor cortex of the mouse brain at 11.7 T. A substantial gain in SNR was observed between CP and RT coils at the same echo‐time. At TE = 16 ms, a greater gain in SNR was observed with the CP coil, as reflected by the signal intensities. All spectra were apodized with a 1 Hz line broadening and a Gaussian multiplication of 0.12 s for display purposes.

Figure 2 illustrates an example of ^1^H LASER spectra acquired from the medial frontal cortex of the mouse brain using a four‐element phased‐array receive coil with a volume resonator for transmission (RT coil) and, a transceiver CP coil. At the TE of 31 ms, which was the shortest achievable with the RT coil due to limited transmit B_1_, the SNR obtained with the CP coil (56.8) exceeded that achieved with the RT coil (13.5) by approximately 4.2‐fold. Furthermore, employing the CP coil with a minimal TE of 16 ms enhanced the SNR even more, reaching 96.1 and thus surpassing the RT coil's performance at TE = 31 ms (13.5) by about 7.1‐fold.

**FIGURE 3:**
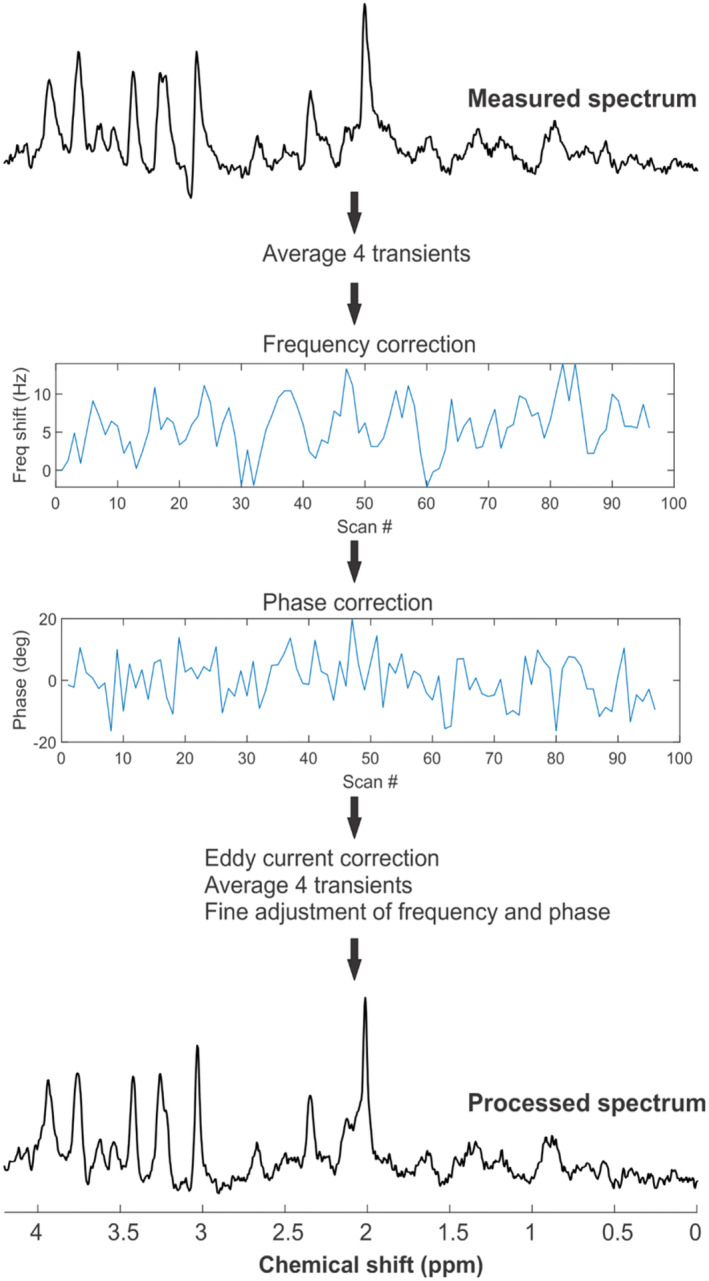
Outline of the post‐acquisition processing steps. The raw MRS data, which contain single‐shot signals for each average from the multi‐element coils, are loaded into MRspa. Signals from each coil are then combined based on the system‐determined phase between the elements. For display purposes, the summed measured spectrum is presented. To increase the SNR of individual shots, FIDs are summed in batches of four transients, followed by frequency correction. Then, phase adjustments are made between shots, followed by eddy current correction. FIDs are again summed in batches of four transients. Finally, fine adjustments of frequency and phase are carried out, and the FIDs are summed to generate the final processed spectra. The spectral linewidth of the processed spectrum is markedly narrower, with minimal phase and baseline issues.

An example of an implemented pre‐processing steps used is shown in Figure 3. Single‐shot spectra were corrected for frequency and phase shifts (due to scanner drift or small animal motion) in addition to performing eddy current correction. The final averaged spectrum (Figure 3) is properly phased with a flat baseline and without any spectral distortion as observed in the measured spectrum. This optimized pre‐processing pipeline will help to obtain reliable concentration estimates.

### 3.2 Distinct In Vivo Metabolite Profile in Primary Motor and Primary Somatosensory Cortex by ^1^H‐MRS

**FIGURE 4:**
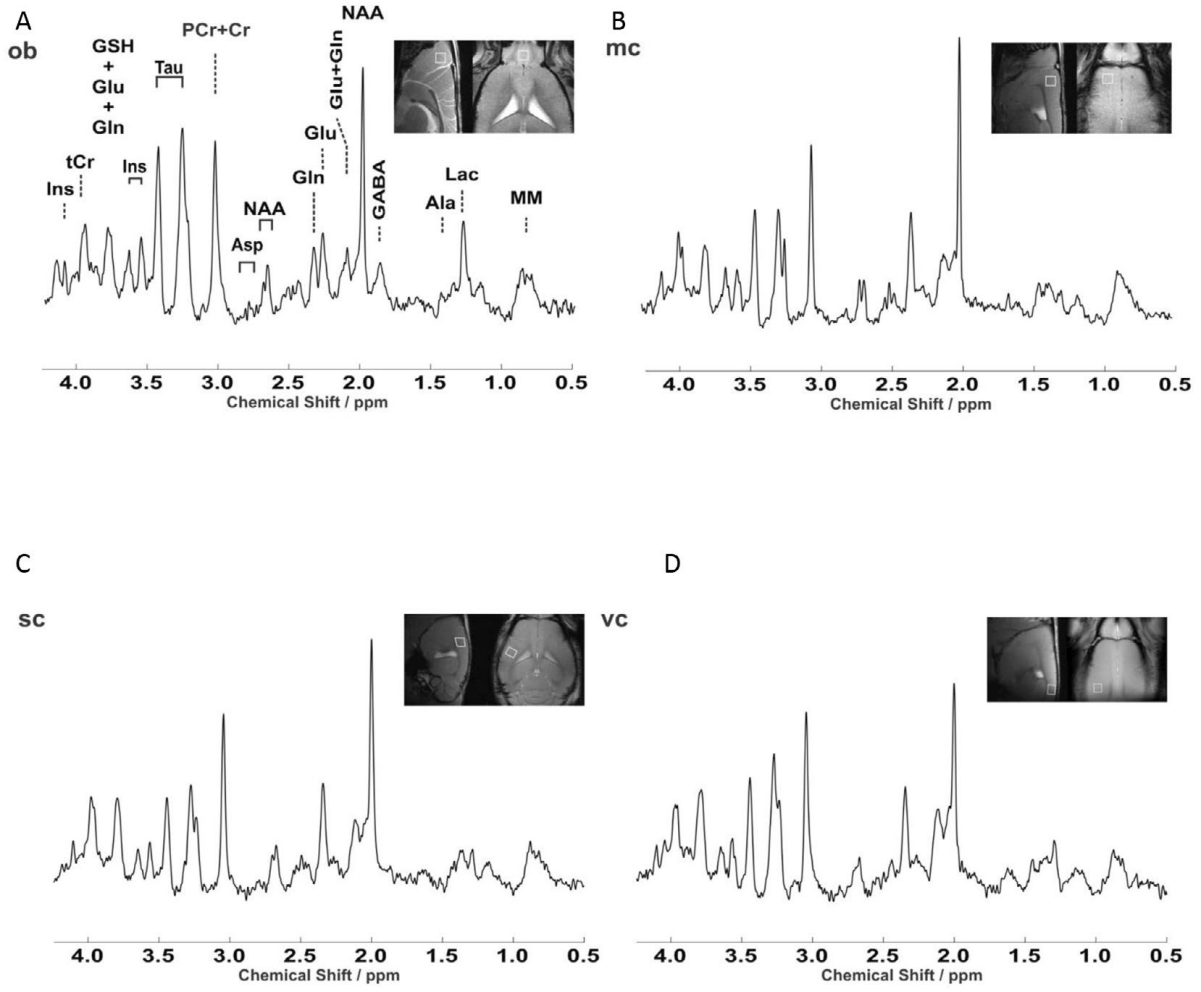
Metabolite fingerprints of different cortical areas. (A) Representative in vivo ^1^H LASER spectrum (TR/TE = 5000/16.74 ms) from the olfactory bulb (ob), indicating the metabolite corresponding to each peak. (B–D) Representative spectra obtained from the primary motor cortex (mop; B), primary somatosensory cortex (sc; C), and primary visual cortex (vc; D). Data were acquired from the mouse brain at 11.7 T. All spectra were apodized with a 1 Hz line broadening and a Gaussian multiplication of 0.12 s for display purposes.

**FIGURE 5:**
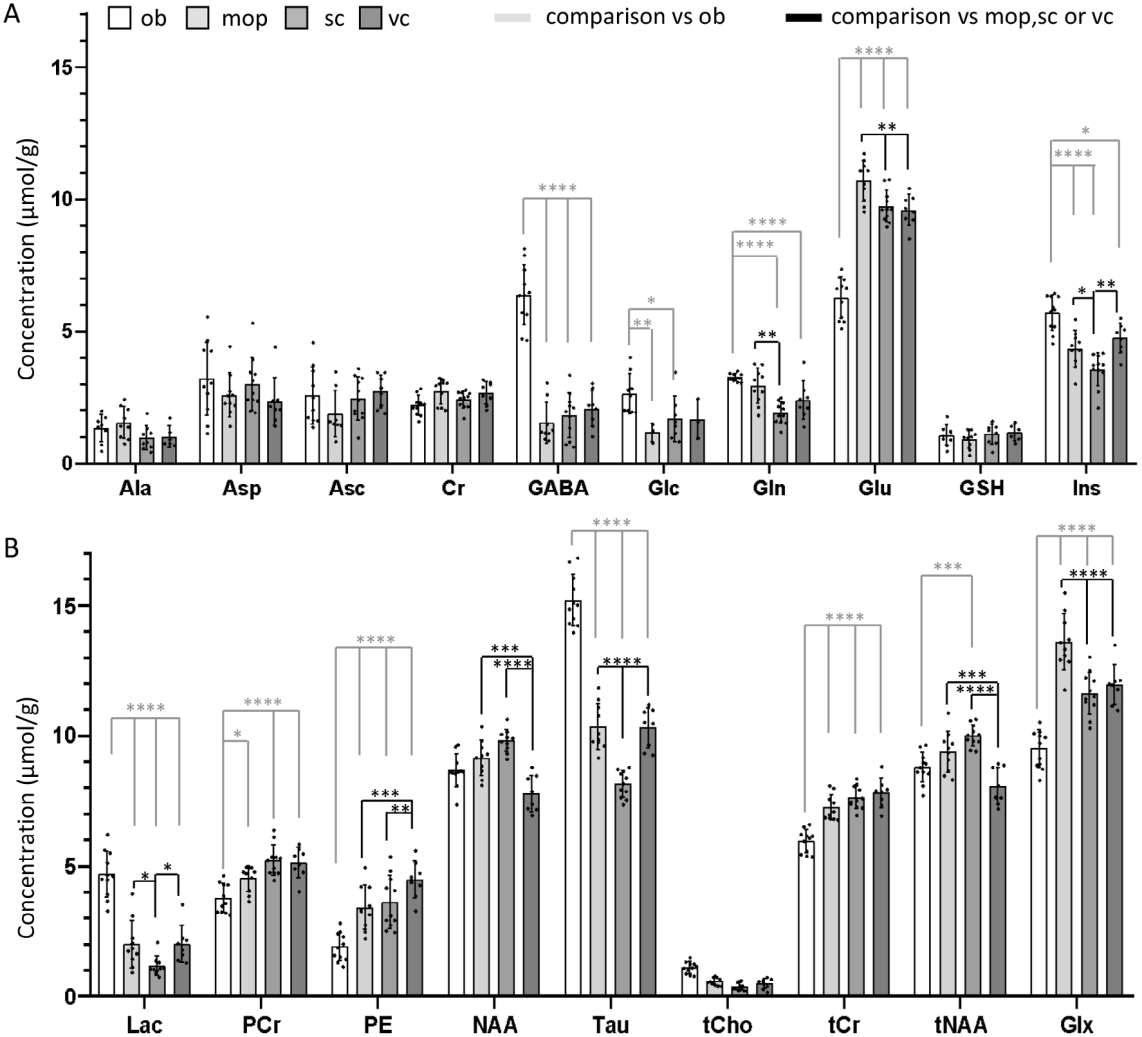
A‐B Direct contrast of the metabolic profiles of motor, somatosensory and visual cortex and olfactor bulb reveals area‐specific metabolite fingerprints. Quantification of the metabolite profiles in the olfactory bulb (ob), primary motor cortex (mop), primary somatosensory cortex, and visual cortex shows that the primary somatosensory cortex displays reduced levels of inositol, lactate, and taurine (A, B). The primary visual cortex shows an increase in phosphoethanolamine (B), while being comparatively more similar to the primary motor cortex than to the primary somatosensory cortex. Sample sizes: Olfactory bulb *n* = 11; Motor cortex *n* = 10; Somatosensory cortex *n* = 11; Visual cortex *n* = 8. ****p < 0.0001; ***p < 0.001; **p < 0.01; *p < 0.05.

We set out to demonstrate that the small VOI enabled by the LASER pipeline allows the identification of distinct metabolite fingerprints from closely opposed cortical areas, such as the motor and somatosensory cortices, as well as from small cortical areas surrounded by other distinct regions. We elected to obtain metabolite fingerprints from the primary motor cortex, primary somatosensory cortex, primary visual cortex, and from a non‐cortical structure, the olfactory bulb. LASER spectra of acceptable quality (FWHM of the water peak not higher than 17 Hz; see methods) were obtained from different VOIs from 8 to 11 mice (three visual cortex VOIs and one motor cortex VOI did not pass the quality‐control criteria; see Section 2; Figure 4). We directly compared visual, motor and somatosensory cortex as well as olfactory bulb to investigate if the small VOI volume enabled the acquisition of distinct spectra profile from different areas. Two‐way ANOVA detected a significant interaction among the metabolite levels and the brain structure (*F*_54,646_ = 34.9; *p* < 0.001). The post hoc comparison (with Holm–Sidak multiple‐comparisons correction) revealed, besides the expected divergence of the olfactory bulb from cortical areas (Table S1), significant differences among the three cortical areas. Statistically significant differences (see Table S2) were detected in the content of glutamine (highest in primary motor cortex; Gln, Figure 5A), glutamate (highest in primary motor cortex; Glu, Figure 5A), *myo*‐inositol (lowest in primary somatosensory cortex; Ins, Figure 5A), phosphoethanolamine (highest in primary visual cortex; PE, Figure 5B), *N*‐acetylaspartate (highest in primary visual cortex; NAA, Figure 4F) and taurine (lowest in primary somatosensory cortex; Tau, Figure 5B). The most pronounced effect size (Cohen's *f*) was observed for taurine (primary somatosensory cortex vs. primary visual cortex *f* = 1.01; primary somatosensory cortex vs. primary motor cortex, *f* = 1.09) and for NAA (primary visual cortex vs. primary somatosensory cortex, *f* = 1.06; primary visual cortex vs. primary motor cortex, *f* = 0.70). The CRLBs corresponding to the fitted metabolites (< 50%) are shown in Figure S1. Combined, these data show that nearby cortical areas can be distinguished by different metabolite fingerprints.

### 3.3 Modification of Metabolite Profile of Primary Motor Cortex During Adulthood

**FIGURE 6:**
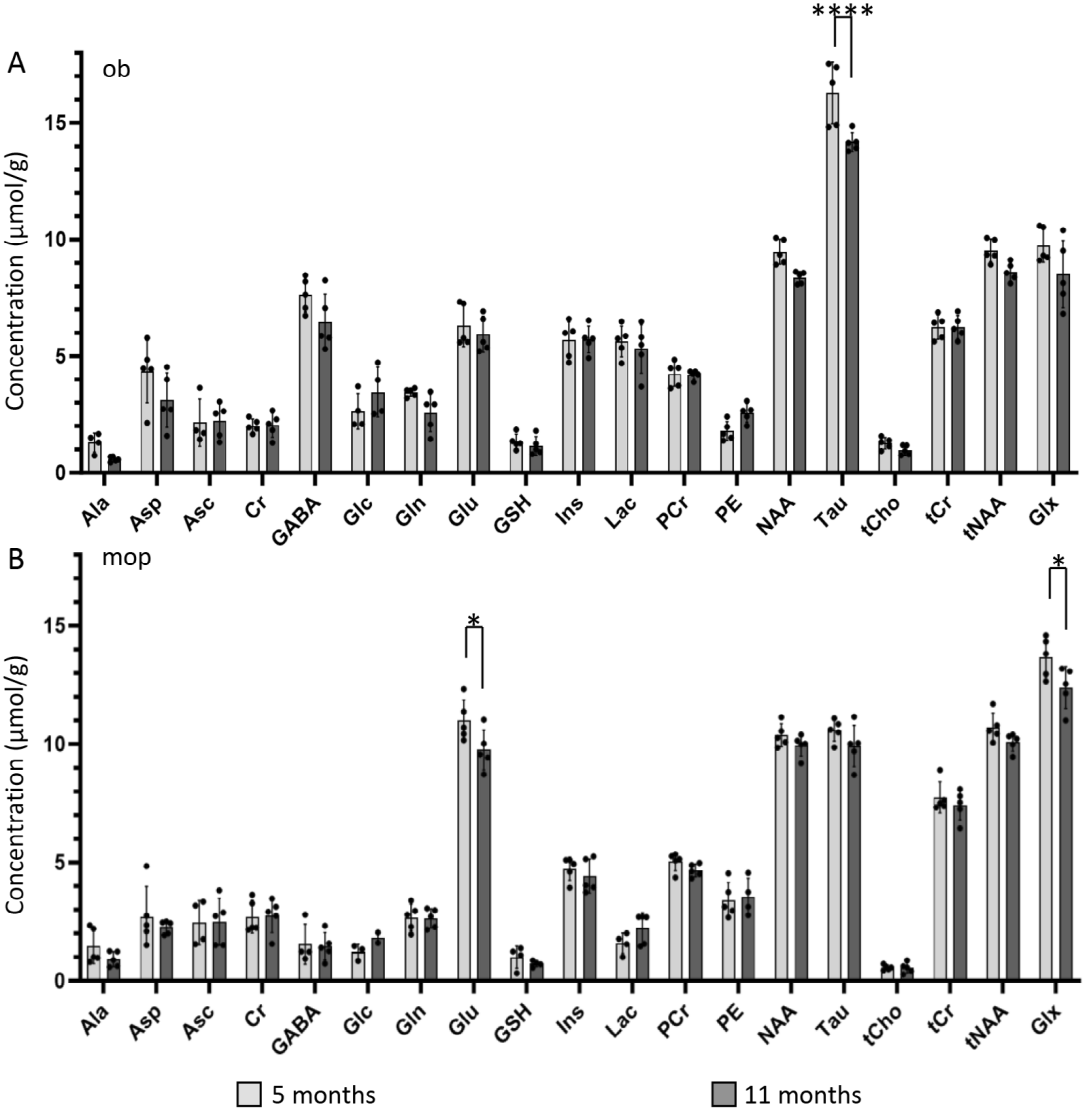
Stability of metabolite fingerprints of brain structures over time. (A) The olfactory bulb (ob) recorded at 5 and 11 months shows comparable profiles, except for a significant decrease in taurine (Tau) concentration. (B) The primary motor cortex (mop) metabolite profile is largely comparable at 5 and 11 months of age, with a decline in glutamate (Glu) and the combined glutamate + glutamine peak in spectra obtained from mice at both time points. Sample sizes: Olfactory bulb *n* = 5; primary motor cortex *n* = 5; VOI = 0.7 μm^3^. ****p < 0.0001; *p < 0.05.

We aimed to determine whether the metabolite profiles of discrete cortical and non‐cortical regions remain stable during adulthood. We considered a VOI of 0.7 μm^3^, ensuring high anatomical homogeneity (i.e., no contamination from nearby, unrelated areas or structures) in the olfactory bulb or primary motor cortex, and acquired spectra at two time points in adulthood, at 5 and 11 months of age. Mixed‐linear model analysis identified a significant age‐dependent effect for olfactory bulb (*F*_1,77_ = 18.37, *p* = 0.0001). Post hoc analysis (with Sidak multiple‐comparisons correction) revealed a significant decline in taurine concentration (16.27 ± 1.31 μmol/g vs. 14.17 ± 0.41 μmol/g at 5 and 11 months, respectively; adjusted *p* = 0.001; Figure 6A).

Most notably, we detected a significant change in the neurochemical profile of the primary motor cortex (mixed linear model analysis *F*_1,70_ = 8.45, *p* = 0.0049): post hoc analysis (Sidak) revealed a significant decrease in both glutamate (10.99 ± 0.86 μmol/g vs. 9.75 ± 0.85 μmol/g at 5 and 11 months, respectively; adjusted *p* = 0.035; Figure 6B) and glutamate + glutamine (13.67 ± 0.84 μmol/g vs. 12.39 ± 0.88 μmol/g at 5 and 11 months, respectively; adjusted *p* = 0.0268; Figure 6B). The CRLBs corresponding to the fitted metabolites are shown in Figure S2. Thus, a small but significant effect of age was detected in the olfactory bulb and primary motor cortex regions.

### 3.4 Sub‐Cortical VOIs Reveal Layer‐Specific Neurochemical Profiles

**FIGURE 7:**
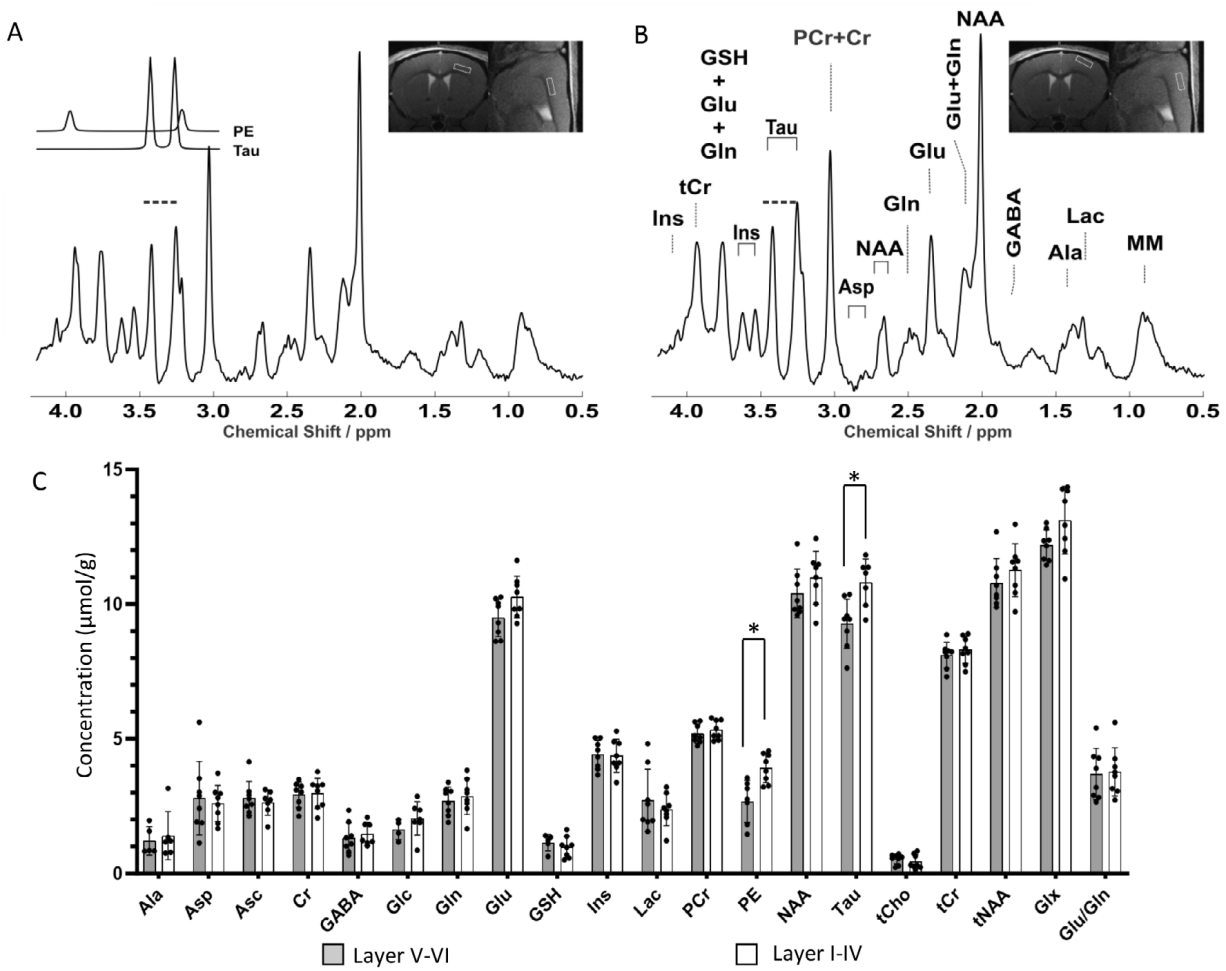
Distinct metabolite fingerprint of upper and deeper cortical layers in primary motor cortex. (A, B) Representative LASER spectra (TR/TE = 5000/17.5 ms, 384 averages) acquired from a 0.8 μL VOI located in lamina V‐VI (A) and lamina I‐IV (B) of the primary motor cortex. The location of the VOI is depicted in the MRI insets (right, A and B). Individual phosphoethanolamine (PE) and taurine (Tau) fits are shown in the inset to (A) (left). All spectra were apodized with a 1 Hz line broadening and a Gaussian multiplication factor of 0.12 s for display purposes. The Tau peaks are highlighted with dotted lines in panels A and B. The PE peak cannot be visually discerned in the spectra because it partially overlaps with the Tau peak (A, inset); it is nevertheless quantified upon deconvolution in LCModel. (C) Quantification of the metabolite fingerprints reveals a significantly higher concentration of taurine (Tau) and phosphoethanolamine (PE) in the upper cortical laminae. Sample sizes: Upper laminae *n* = 8; Lower laminae *n* = 8. *p < 0.05.

Finally, we utilized the small VOI enabled by LASER to resolve the metabolic profile of sub‐compartments within a single cortical area, specifically the upper and lower laminae of the primary motor cortex. High‐quality LASER spectra were acquired from the upper and lower cortical layers of the primary motor cortex (Figure 7). A two‐way ANOVA revealed a significant difference in the metabolite profile (19 analytes) between upper and lower cortical layers (*F*_1,263_ = 12.21, *p* = 0.0006). Post hoc analysis (Sidak's multiple‐comparisons) revealed a difference in taurine and PE, which were both significantly more abundant in upper layers (taurine: 10.32 ± 0.84 μmol/g in upper layers vs. 9.25 ± 0.98 μmol/g in deeper layers, *p* = 0.0012; PE: 3.91 ± 0.54 μmol/g in upper layers vs. 2.67 ± 0.78 μmol/g in deeper layers, *p* = 0.0211; Figure 7C). Similar results were obtained when a two‐stage linear step‐up procedure (Benjamini, Krieger and Yekutieli) was applied to account for False Discovery Rate (*q*) =0.05. The CRLBs corresponding to the fitted metabolites are shown in Figure S3. Thus, our ^1^H‐MRS pipeline was able to distinguish metabolite fingerprints in the upper and lower cortical layers of the primary motor cortex.

## 4 Discussion

Here we have demonstrated that a combination of cutting‐edge hardware solution (high‐field magnet, low‐noise cryocoil RF coil), pulse design (LASER) and a dedicated off‐line pre‐processing protocol can increase the sensitivity of small‐animal MRS to quantify 19 distinct metabolites from sub‐microliter volumes in vivo. This so far unreached spatial and spectral fidelity of the proposed MRS pipeline enables distinctive metabolite fingerprints of different but nearby cortical areas and of metabolite fingerprints of upper and lower layers of the same cortical area.

The implementation of sub‐microliter MRS reveals that three functionally diverse cortical areas, namely the primary motor cortex, primary somatosensory cortex, and primary visual cortex, display intriguing metabolite differences and are also significantly different from the olfactory bulb, as previously reported (Florian et al. 1996). Despite their close anatomical proximity and strong functional interaction (Sreenivasan et al. 2016), the primary motor cortex and primary somatosensory cortex are distinguished by lower concentrations of glutamate, glutamine, *myo*‐inositol, lactate, and taurine in the primary somatosensory cortex (Figure 4E,F). On the other hand, the primary visual cortex displays levels of taurine and *myo*‐inositol similar to those in the primary motor cortex and levels of glutamate similar to those in the primary somatosensory cortex. Some of these neurochemical signatures could be attributed to differences in cytoarchitectonics: the lower representation of taurine in the primary somatosensory cortex compared to the primary visual cortex has been previously attributed to the lower density of taurine‐positive interneurons in the former (Kritzer et al. 1992). Likewise, since glutamine synthase is uniquely expressed in astrocytes (Anlauf and Derouiche 2013), the regional differences in glutamine may reflect the number or the metabolic activity of these glial cells. Indeed, the Allen Brain Atlas expression dataset reveals a higher expression of glutamine synthase in primary motor cortex vs. primary somatosensory cortex and primary visual cortex and histological data show that the density of non‐neuronal (mostly glial) cells is highest in primary motor cortex and lowest in S1 (Herculano‐Houzel, Watson, and Paxinos 2013). Conversely, primary visual cortex displays the lowest concentration of NAA (and tNAA) among the three areas tested, and the highest concentration of PE. Once again, these neurochemical profiles are in agreement with the cytoarchitectonics of primary visual cortex: PE constitutes up to 25% of lipid components of cell membranes (Vance 2015) and it is considered a proxy of cellular density (Duarte et al. 2012). As primary visual cortex is the cortical area with highest neuronal density compared to primary motor cortex and primary somatosensory cortex (Herculano‐Houzel, Watson, and Paxinos 2013), the relative elevation of PE may reflect the higher neuronal density. On the other hand, NAA has been previously shown to be detectable in all cortical neurons but to be highly enriched in lamina five neurons (Moffett, Namboodiri, and Neale 1993), which is comparatively less represented in primary visual cortex than in primary motor cortex (Balaram and Kaas 2014; Barbas and García‐Cabezas 2015). Furthermore, the higher glutamate concentration in primary motor cortex is in agreement with the higher density of synapses in motor cortex compared to visual cortex (Schüz and Palm 1989). Thus, sub‐microliter MRS identifies metabolite signatures associated with different cellular and synaptic architectures of different cortical areas.

We also confirmed that during adult age (5 and 11 months), the metabolic fingerprints of different areas is largely stable. The small decrease in taurine in olfactory bulb and the small decrease in glutamate and glutamate +glutamine peaks in primary motor cortex are in overall agreement with previously reported trends (Duarte, Do, and Gruetter 2014; Zhu et al. 2019). Of note, we have identified a significant loss in glutamate whereas previously statistical significance was reached only for more advanced ages and more substantial losses (Duarte, Do, and Gruetter 2014), indicating that our pipeline is sensitive to metabolite changes occurring during adulthood.

Finally, we have exploited the established protocol small VOI to obtain in vivo metabolite signatures of superficial and deep layers in the mouse cortex; this constitutes a substantial advancement when compared to previous determinations of MRS which had to include not only the whole cortical thickness but often two cortical areas from opposite hemispheres (Duarte, Do, and Gruetter 2014). Notably, the metabolic profile of the superficial and deep layers are distinguished by higher concentrations of taurine and phosphoethanolamine in the superficial layers compared to deep layers. These findings are in agreement with the higher density of taurine‐positive cells in upper vs. lower cortical layers demonstrated by immunohistochemical methods (Kritzer et al. 1992; Pow et al. 2002). Likewise, PE levels, which are considered an estimate of cellularity, and/or mitochondrial content (Duarte et al. 2012), are in agreement with the higher neuronal density and higher mitochondrial content measured in upper layers by histological techniques (Keller, Erö, and Markram 2018; Santuy et al. 2018). Notably, if PE was mainly influenced by myelination, it would be higher in deeper layers, which are more enriched with myelinated fibers (Santuy et al. 2018). Thus, sub‐microlitre MRS appears able to resolve details of the cellular composition of cortical lamination.

Our MRS pipeline provides very high sensitivity, enabling both the precise characterization of multiple metabolites and the use of sub‐microliter volumes of interest (VOIs). Due to the large bandwidth of the pulses used in LASER, the chemical shift displacement error (CSDE) was minimal (3.6%/ppm), translating to a 0.1 mm displacement in all three directions for the 3 ppm chemical shift region. Therefore, this technique enables the characterization of metabolic profiles of neuronal populations subject to injury or vulnerability to disease, free from contamination or dilution effects from nearby areas.

Notably, the metabolite concentrations expected in larger VOIs are not necessarily a linear average of those in smaller VOIs, as the shimming profile and partial‐volume effects are differently affected by surrounding heterogeneous structures in the two VOI sizes. However, some limitations of the current technique should be acknowledged. First, the acquisition time remains over 30 min per VOI, necessitating prolonged anesthesia for acquiring multi‐voxel datasets. Although randomizing VOI acquisition may mitigate biases, the stability of metabolite levels under different types and durations of anesthesia requires further investigation. Second, the performance of the CryoProbe diminishes with increasing distance of the VOI from the probe, thereby preventing homogeneous resolution throughout the brain.

In conclusion, our work presents a novel framework for sub‐microliter in vivo rodent spectroscopy that combines advanced processing techniques with state‐of‐the‐art hardware innovations. By addressing key challenges in spectral resolution, sensitivity, and sample stability, we propel the field toward more precise and insightful neuroimaging studies. Future endeavors building upon our methodology hold immense promise for advancing our understanding of brain function and dysfunction, ultimately benefiting both basic neuroscience research and clinical applications.

## Author Contributions

**Alireza Abaei:** conceptualization, investigation, writing – original draft, methodology, validation, writing – review and editing, visualization, formal analysis, data curation. **Dinesh K. Deelchand:** conceptualization, writing – original draft, methodology, validation, writing – review and editing, formal analysis, software, data curation. **Jan Kassubek:** supervision. **Francescois Roselli:** conceptualization, investigation, writing – original draft, writing – review and editing, formal analysis, data curation. **Volker Rasche:** funding acquisition, supervision.

## Conflicts of Interest

The authors declare no conflicts of interest.

## Supporting information


**Table S1.** Details of the statistical analysis (two‐way ANOVA with post hoc Sidak) comparing absolute concentrations (μmol/g) of the detected analytes.
**Table S2:** Details of the statistical analysis (two‐way ANOVA with post hoc Sidak) comparing the spectra of different cortical regions (excluding the OB).
**Figure S1:** Mean Cramér–Rao Lower Bound (CRLB) estimates for the metabolites quantified in Figure 5, expressed in relative units (%). Only metabolites with CRLB < 50% were considered for analysis.
**Figure S2:** Mean Cramér–Rao Lower Bound (CRLB) estimates for the metabolites quantified in Figure 6, expressed in relative units (%). Only metabolites with CRLB < 50% were considered for analysis.
**Figure S3:** Mean Cramér–Rao Lower Bound (CRLB) estimates for the metabolites quantified in Figure 3, expressed in relative units (%). Only metabolites with CRLB < 50% were considered for analysis.

## Data Availability

The individual spectra and related data are available from the corresponding author upon reasonable request.
