# Supplementary material for: Sub‐Microliter 1H Magnetic Resonance Spectroscopy for In Vivo High‐Spatial Resolution Metabolite Quantification in the Mouse Brain
Source: J Neurochem. 2025 Jan 18;169(1):e16303. doi: 10.1111/jnc.16303 (PMC11742661; doi:10.1111/jnc.16303)

Supplementary information

Sub-microliter ^1H magnetic resonance spectroscopy for in vivo high-spatial resolution metabolite quantification in the mouse brain

Authors: Alireza Abaei[1], Dinesh K. Deelchand[2], Jan Kassubek[3,4], Francesco Roselli[3,4], Volker Rasche[1,5]

Affiliations:
1. Core Facility Small Animal MRI, Ulm University, Ulm, Germany
2. Center for Magnetic Resonance Research, University of Minnesota Minneapolis, MN, USA
3. Department of Neurology, Ulm University, Ulm, Germany
4. German Center for Neurodegenerative Diseases (DZNE)-Ulm, Germany
5. Department of Internal Medicine II, Ulm University Medical Center, Ulm, Germany

Supplementary Table 1: details of the statistical analysis (two-way ANOVA with post-hoc Sidak) comparing absolute concentrations (µmol/g) of the detected analytes

Test details	Predicted (LS) mean 1	Predicted (LS) mean 2	Predicted (LS) mean diff.	SE of diff.	N1	N2	q	DF
Ala								
MOp vs. SS	1.476	0.9592	0.5166	0.3283	10	9	2.225	700.0
MOp vs. V1	1.476	0.9997	0.4761	0.3690	10	6	1.825	700.0
MOp vs. Ob	1.476	1.320	0.1561	0.3389	10	8	0.6511	700.0
SS vs. V1	0.9592	0.9997	-0.04044	0.3766	9	6	0.1519	700.0
SS vs. Ob	0.9592	1.320	-0.3605	0.3472	9	8	1.468	700.0
V1 vs. Ob	0.9997	1.320	-0.3201	0.3859	6	8	1.173	700.0
Asp								
MOp vs. SS	2.751	2.997	-0.2463	0.3047	11	11	1.143	700.0
MOp vs. V1	2.751	2.313	0.4383	0.3320	11	8	1.867	700.0
MOp vs. Ob	2.751	3.194	-0.4430	0.3047	11	11	2.056	700.0
SS vs. V1	2.997	2.313	0.6846	0.3320	11	8	2.916	700.0
SS vs. Ob	2.997	3.194	-0.1967	0.3047	11	11	0.9131	700.0
V1 vs. Ob	2.313	3.194	-0.8813	0.3320	8	11	3.754	700.0
Asc								
MOp vs. SS	1.999	2.440	-0.4408	0.3212	9	11	1.941	700.0
MOp vs. V1	1.999	2.711	-0.7112	0.3472	9	8	2.897	700.0
MOp vs. Ob	1.999	2.563	-0.5641	0.3283	9	10	2.430	700.0
SS vs. V1	2.440	2.711	-0.2704	0.3320	11	8	1.152	700.0
SS vs. Ob	2.440	2.563	-0.1233	0.3122	11	10	0.5585	700.0
V1 vs. Ob	2.711	2.563	0.1471	0.3389	8	10	0.6138	700.0
Cr								
MOp vs. SS	2.786	2.412	0.3739	0.3047	11	11	1.735	700.0
MOp vs. V1	2.786	2.669	0.1169	0.3320	11	8	0.4980	700.0
MOp vs. Ob	2.786	2.192	0.5944	0.3047	11	11	2.759	700.0
SS vs. V1	2.412	2.669	-0.2570	0.3320	11	8	1.095	700.0
SS vs. Ob	2.412	2.192	0.2205	0.3047	11	11	1.023	700.0
V1 vs. Ob	2.669	2.192	0.4775	0.3320	8	11	2.034	700.0
GABA								
MOp vs. SS	1.518	1.806	-0.2882	0.3212	9	11	1.269	700.0
MOp vs. V1	1.518	2.061	-0.5436	0.3472	9	8	2.214	700.0
MOp vs. Ob	1.518	6.367	-4.850	0.3212	9	11	21.35	700.0
SS vs. V1	1.806	2.061	-0.2554	0.3320	11	8	1.088	700.0
SS vs. Ob	1.806	6.367	-4.561	0.3047	11	11	21.17	700.0
V1 vs. Ob	2.061	6.367	-4.306	0.3320	8	11	18.34	700.0
Glc								
MOp vs. SS	1.216	1.675	-0.4597	0.4376	4	8	1.486	700.0
MOp vs. V1	1.216	1.657	-0.4415	0.5458	4	3	1.144	700.0
MOp vs. Ob	1.216	2.626	-1.411	0.4294	4	9	4.647	700.0
SS vs. V1	1.675	1.657	0.01825	0.4838	8	3	0.05335	700.0
SS vs. Ob	1.675	2.626	-0.9511	0.3472	8	9	3.874	700.0
V1 vs. Ob	1.657	2.626	-0.9693	0.4764	3	9	2.878	700.0
Gln								
MOp vs. SS	2.893	1.901	0.9918	0.3047	11	11	4.604	700.0
MOp vs. V1	2.893	2.380	0.5129	0.3320	11	8	2.185	700.0
MOp vs. Ob	2.893	3.249	-0.3565	0.3047	11	11	1.654	700.0
SS vs. V1	1.901	2.380	-0.4789	0.3320	11	8	2.040	700.0
SS vs. Ob	1.901	3.249	-1.348	0.3047	11	11	6.258	700.0
V1 vs. Ob	2.380	3.249	-0.8694	0.3320	8	11	3.703	700.0
Glu								

MOp vs. SS	10.57	9.734	0.8392	0.3047	11	11	3.895	700.0
MOp vs. V1	10.57	9.580	0.9936	0.3320	11	8	4.232	700.0
MOp vs. Ob	10.57	6.257	4.316	0.3047	11	11	20.03	700.0
SS vs. V1	9.734	9.580	0.1544	0.3320	11	8	0.6575	700.0
SS vs. Ob	9.734	6.257	3.477	0.3047	11	11	16.14	700.0
V1 vs. Ob	9.580	6.257	3.323	0.3320	8	11	14.15	700.0
GSH								
MOp vs. SS	0.9300	1.095	-0.1649	0.3389	10	8	0.6879	700.0
MOp vs. V1	0.9300	1.161	-0.2307	0.3690	10	6	0.8840	700.0
MOp vs. Ob	0.9300	1.051	-0.1206	0.3283	10	9	0.5193	700.0
SS vs. V1	1.095	1.161	-0.06579	0.3859	8	6	0.2411	700.0
SS vs. Ob	1.095	1.051	0.04432	0.3472	8	9	0.1805	700.0
V1 vs. Ob	1.161	1.051	0.1101	0.3766	6	9	0.4135	700.0
Ins								
MOp vs. SS	4.367	3.543	0.8241	0.3047	11	11	3.825	700.0
MOp vs. V1	4.367	4.730	-0.3625	0.3320	11	8	1.544	700.0
MOp vs. Ob	4.367	5.698	-1.331	0.3047	11	11	6.177	700.0
SS vs. V1	3.543	4.730	-1.187	0.3320	11	8	5.054	700.0
SS vs. Ob	3.543	5.698	-2.155	0.3047	11	11	10.00	700.0
V1 vs. Ob	4.730	5.698	-0.9683	0.3320	8	11	4.124	700.0
Lac								
MOp vs. SS	2.019	1.181	0.8377	0.3196	10	10	3.707	700.0
MOp vs. V1	2.019	2.019	-0.0006750	0.3389	10	8	0.002816	700.0
MOp vs. Ob	2.019	4.702	-2.684	0.3122	10	11	12.16	700.0
SS vs. V1	1.181	2.019	-0.8384	0.3389	10	8	3.498	700.0
SS vs. Ob	1.181	4.702	-3.521	0.3122	10	11	15.95	700.0
V1 vs. Ob	2.019	4.702	-2.683	0.3320	8	11	11.43	700.0
PCr								
MOp vs. SS	4.568	5.231	-0.6632	0.3047	11	11	3.078	700.0
MOp vs. V1	4.568	5.152	-0.5845	0.3320	11	8	2.490	700.0
MOp vs. Ob	4.568	3.773	0.7948	0.3047	11	11	3.689	700.0
SS vs. V1	5.231	5.152	0.07866	0.3320	11	8	0.3350	700.0
SS vs. Ob	5.231	3.773	1.458	0.3047	11	11	6.767	700.0
V1 vs. Ob	5.152	3.773	1.379	0.3320	8	11	5.875	700.0
PE								
MOp vs. SS	3.375	3.616	-0.2405	0.3047	11	11	1.116	700.0
MOp vs. V1	3.375	4.491	-1.116	0.3320	11	8	4.752	700.0
MOp vs. Ob	3.375	1.913	1.463	0.3047	11	11	6.789	700.0
SS vs. V1	3.616	4.491	-0.8751	0.3320	11	8	3.728	700.0
SS vs. Ob	3.616	1.913	1.703	0.3047	11	11	7.905	700.0
V1 vs. Ob	4.491	1.913	2.578	0.3320	8	11	10.98	700.0
NAA								
MOp vs. SS	9.169	9.830	-0.6611	0.3047	11	11	3.068	700.0
MOp vs. V1	9.169	7.792	1.377	0.3320	11	8	5.866	700.0
MOp vs. Ob	9.169	8.674	0.4945	0.3047	11	11	2.295	700.0
SS vs. V1	9.830	7.792	2.038	0.3320	11	8	8.682	700.0
SS vs. Ob	9.830	8.674	1.156	0.3047	11	11	5.363	700.0
V1 vs. Ob	7.792	8.674	-0.8828	0.3320	8	11	3.760	700.0
Tau								
MOp vs. SS	10.35	8.160	2.193	0.3047	11	11	10.18	700.0
MOp vs. V1	10.35	10.31	0.04356	0.3320	11	8	0.1855	700.0
MOp vs. Ob	10.35	15.21	-4.856	0.3047	11	11	22.54	700.0
SS vs. V1	8.160	10.31	-2.149	0.3320	11	8	9.155	700.0
SS vs. Ob	8.160	15.21	-7.049	0.3047	11	11	32.72	700.0
V1 vs. Ob	10.31	15.21	-4.899	0.3320	8	11	20.87	700.0

tCho								
MOp vs. SS	0.5992	0.3925	0.2067	0.3047	11	11	0.9595	700.0
MOp vs. V1	0.5992	0.5070	0.09218	0.3320	11	8	0.3926	700.0
MOp vs. Ob	0.5992	1.118	-0.5193	0.3047	11	11	2.410	700.0
SS vs. V1	0.3925	0.5070	-0.1145	0.3320	11	8	0.4879	700.0
SS vs. Ob	0.3925	1.118	-0.7260	0.3047	11	11	3.370	700.0
V1 vs. Ob	0.5070	1.118	-0.6115	0.3320	8	11	2.604	700.0
tCr								
MOp vs. SS	7.354	7.643	-0.2894	0.3047	11	11	1.343	700.0
MOp vs. V1	7.354	7.821	-0.4677	0.3320	11	8	1.992	700.0
MOp vs. Ob	7.354	5.964	1.389	0.3047	11	11	6.449	700.0
SS vs. V1	7.643	7.821	-0.1784	0.3320	11	8	0.7598	700.0
SS vs. Ob	7.643	5.964	1.679	0.3047	11	11	7.792	700.0
V1 vs. Ob	7.821	5.964	1.857	0.3320	8	11	7.910	700.0
tNAA								
MOp vs. SS	9.435	10.03	-0.5910	0.3047	11	11	2.743	700.0
MOp vs. V1	9.435	8.084	1.352	0.3320	11	8	5.757	700.0
MOp vs. Ob	9.435	8.801	0.6340	0.3047	11	11	2.943	700.0
SS vs. V1	10.03	8.084	1.943	0.3320	11	8	8.274	700.0
SS vs. Ob	10.03	8.801	1.225	0.3047	11	11	5.686	700.0
V1 vs. Ob	8.084	8.801	-0.7175	0.3320	8	11	3.056	700.0
Glx								
MOp vs. SS	13.47	11.63	1.831	0.3047	11	11	8.497	700.0
MOp vs. V1	13.47	11.96	1.506	0.3320	11	8	6.416	700.0
MOp vs. Ob	13.47	9.506	3.960	0.3047	11	11	18.38	700.0
SS vs. V1	11.63	11.96	-0.3243	0.3320	11	8	1.381	700.0
SS vs. Ob	11.63	9.506	2.129	0.3047	11	11	9.882	700.0
V1 vs. Ob	11.96	9.506	2.453	0.3320	8	11	10.45	700.0
Glu/Gln								
MOp vs. SS	3.831	5.346	-1.515	0.3047	11	11	7.032	700.0
MOp vs. V1	3.831	4.388	-0.5570	0.3320	11	8	2.372	700.0
MOp vs. Ob	3.831	1.934	1.897	0.3047	11	11	8.805	700.0
SS vs. V1	5.346	4.388	0.9581	0.3320	11	8	4.081	700.0
SS vs. Ob	5.346	1.934	3.412	0.3047	11	11	15.84	700.0
V1 vs. Ob	4.388	1.934	2.454	0.3320	8	11	10.45	700.0

Supplementary Table 2: details of the statistical analysis comparing the spectra of different cortical region (excluding the OB).

Test details	Predicted (LS) mean 1	Predicted (LS) mean 2	Predicted (LS) mean diff.	SE of diff.	N1	N2	q	DF
Ala								
MOP vs. SS	1.476	0.9592	0.5166	0.3258	10	9	2.243	508.0
MOP vs. V1	1.476	0.9997	0.4761	0.3661	10	6	1.839	508.0
SS vs. V1	0.9592	0.9997	-0.04044	0.3737	9	6	0.1531	508.0
Asp								
MOP vs. SS	2.751	2.997	-0.2463	0.3023	11	11	1.152	508.0
MOP vs. V1	2.751	2.313	0.4383	0.3294	11	8	1.882	508.0
SS vs. V1	2.997	2.313	0.6846	0.3294	11	8	2.939	508.0
Asc								
MOP vs. SS	1.999	2.440	-0.4408	0.3187	9	11	1.956	508.0
MOP vs. V1	1.999	2.711	-0.7112	0.3445	9	8	2.919	508.0
SS vs. V1	2.440	2.711	-0.2704	0.3294	11	8	1.161	508.0
Cr								
MOP vs. SS	2.786	2.412	0.3739	0.3023	11	11	1.749	508.0
MOP vs. V1	2.786	2.669	0.1169	0.3294	11	8	0.5019	508.0
SS vs. V1	2.412	2.669	-0.2570	0.3294	11	8	1.103	508.0
GABA								
MOP vs. SS	1.518	1.806	-0.2882	0.3187	9	11	1.279	508.0
MOP vs. V1	1.518	2.061	-0.5436	0.3445	9	8	2.231	508.0
SS vs. V1	1.806	2.061	-0.2554	0.3294	11	8	1.096	508.0
Glc								
MOP vs. SS	1.215	1.675	-0.4598	0.4342	4	8	1.498	508.0
MOP vs. V1	1.215	1.657	-0.4415	0.5415	4	3	1.153	508.0
SS vs. V1	1.675	1.657	0.01825	0.4800	8	3	0.05377	508.0
Gln								
MOP vs. SS	2.893	1.901	0.9918	0.3023	11	11	4.640	508.0
MOP vs. V1	2.893	2.380	0.5129	0.3294	11	8	2.202	508.0
SS vs. V1	1.901	2.380	-0.4789	0.3294	11	8	2.056	508.0
Glu								
MOP vs. SS	10.57	9.734	0.8392	0.3023	11	11	3.926	508.0
MOP vs. V1	10.57	9.580	0.9936	0.3294	11	8	4.265	508.0
SS vs. V1	9.734	9.580	0.1544	0.3294	11	8	0.6627	508.0
GSH								
MOP vs. SS	0.9300	1.095	-0.1649	0.3363	10	8	0.6933	508.0
MOP vs. V1	0.9300	1.161	-0.2307	0.3661	10	6	0.8910	508.0
SS vs. V1	1.095	1.161	-0.06579	0.3829	8	6	0.2430	508.0
Ins								
MOP vs. SS	4.367	3.543	0.8241	0.3023	11	11	3.855	508.0
MOP vs. V1	4.367	4.730	-0.3625	0.3294	11	8	1.556	508.0
SS vs. V1	3.543	4.730	-1.187	0.3294	11	8	5.094	508.0
Lac								
MOP vs. SS	2.019	1.181	0.8377	0.3171	10	10	3.736	508.0
MOP vs. V1	2.019	2.019	-0.0006750	0.3363	10	8	0.002838	508.0
SS vs. V1	1.181	2.019	-0.8384	0.3363	10	8	3.525	508.0
PCr								
MOP vs. SS	4.568	5.231	-0.6632	0.3023	11	11	3.102	508.0
MOP vs. V1	4.568	5.152	-0.5845	0.3294	11	8	2.509	508.0
SS vs. V1	5.231	5.152	0.07866	0.3294	11	8	0.3377	508.0

PE								
MOP vs. SS	3.375	3.616	-0.2405	0.3023	11	11	1.125	508.0
MOP vs. V1	3.375	4.491	-1.116	0.3294	11	8	4.789	508.0
SS vs. V1	3.616	4.491	-0.8751	0.3294	11	8	3.757	508.0
NAA								
MOP vs. SS	9.169	9.830	-0.6611	0.3023	11	11	3.093	508.0
MOP vs. V1	9.169	7.792	1.377	0.3294	11	8	5.912	508.0
SS vs. V1	9.830	7.792	2.038	0.3294	11	8	8.750	508.0
Tau								
MOP vs. SS	10.35	8.160	2.193	0.3023	11	11	10.26	508.0
MOP vs. V1	10.35	10.31	0.04356	0.3294	11	8	0.1870	508.0
SS vs. V1	8.160	10.31	-2.149	0.3294	11	8	9.227	508.0
tCho								
MOP vs. SS	0.5992	0.3925	0.2067	0.3023	11	11	0.9671	508.0
MOP vs. V1	0.5992	0.5070	0.09218	0.3294	11	8	0.3957	508.0
SS vs. V1	0.3925	0.5070	-0.1145	0.3294	11	8	0.4917	508.0
tCr								
MOP vs. SS	7.354	7.643	-0.2894	0.3023	11	11	1.354	508.0
MOP vs. V1	7.354	7.821	-0.4677	0.3294	11	8	2.008	508.0
SS vs. V1	7.643	7.821	-0.1784	0.3294	11	8	0.7657	508.0
tNAA								
MOP vs. SS	9.435	10.03	-0.5910	0.3023	11	11	2.765	508.0
MOP vs. V1	9.435	8.084	1.352	0.3294	11	8	5.802	508.0
SS vs. V1	10.03	8.084	1.943	0.3294	11	8	8.339	508.0
Glx								
MOP vs. SS	13.47	11.63	1.831	0.3023	11	11	8.564	508.0
MOP vs. V1	13.47	11.96	1.506	0.3294	11	8	6.467	508.0
SS vs. V1	11.63	11.96	-0.3243	0.3294	11	8	1.392	508.0
Glu/Gln								
MOP vs. SS	3.831	5.346	-1.515	0.3023	11	11	7.087	508.0
MOP vs. V1	3.831	4.388	-0.5570	0.3294	11	8	2.391	508.0
SS vs. V1	5.346	4.388	0.9581	0.3294	11	8	4.113	508.0

Supplementary Figure 1: Mean Cramér–Rao Lower Bound (CRLB) estimates for the metabolites quantified in Figure 5, expressed in relative units (%). Only metabolites with CRLB < 50% were considered for analysis.

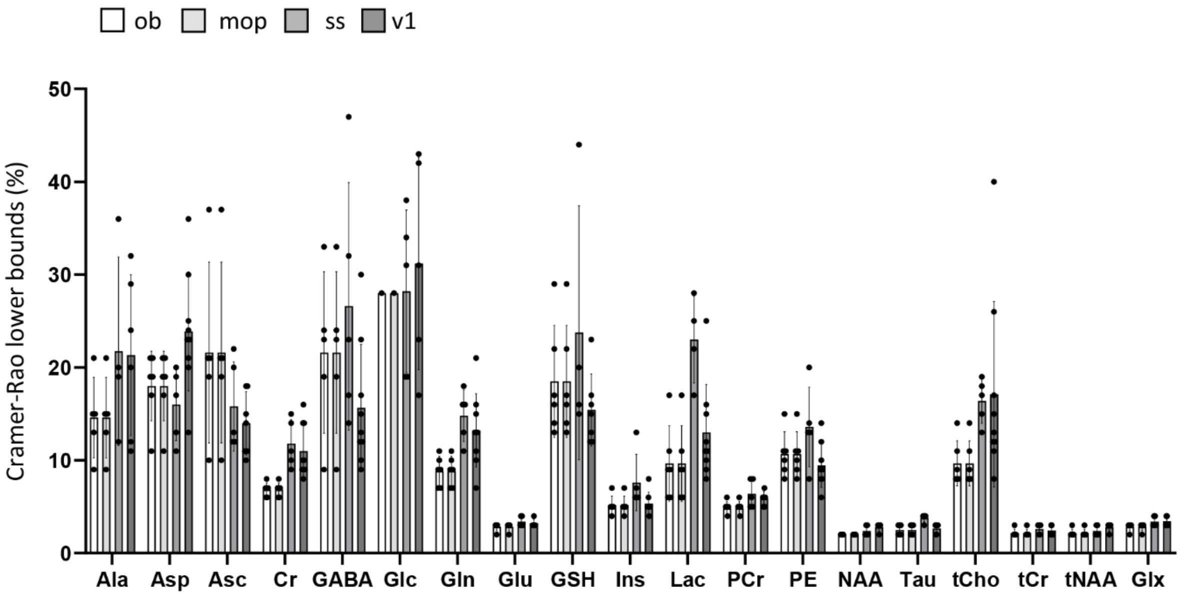

Supplementary Figure 2: Mean Cramér–Rao Lower Bound (CRLB) estimates for the metabolites quantified in Figure 6, expressed in relative units (%). Only metabolites with CRLB < 50% were considered for analysis.

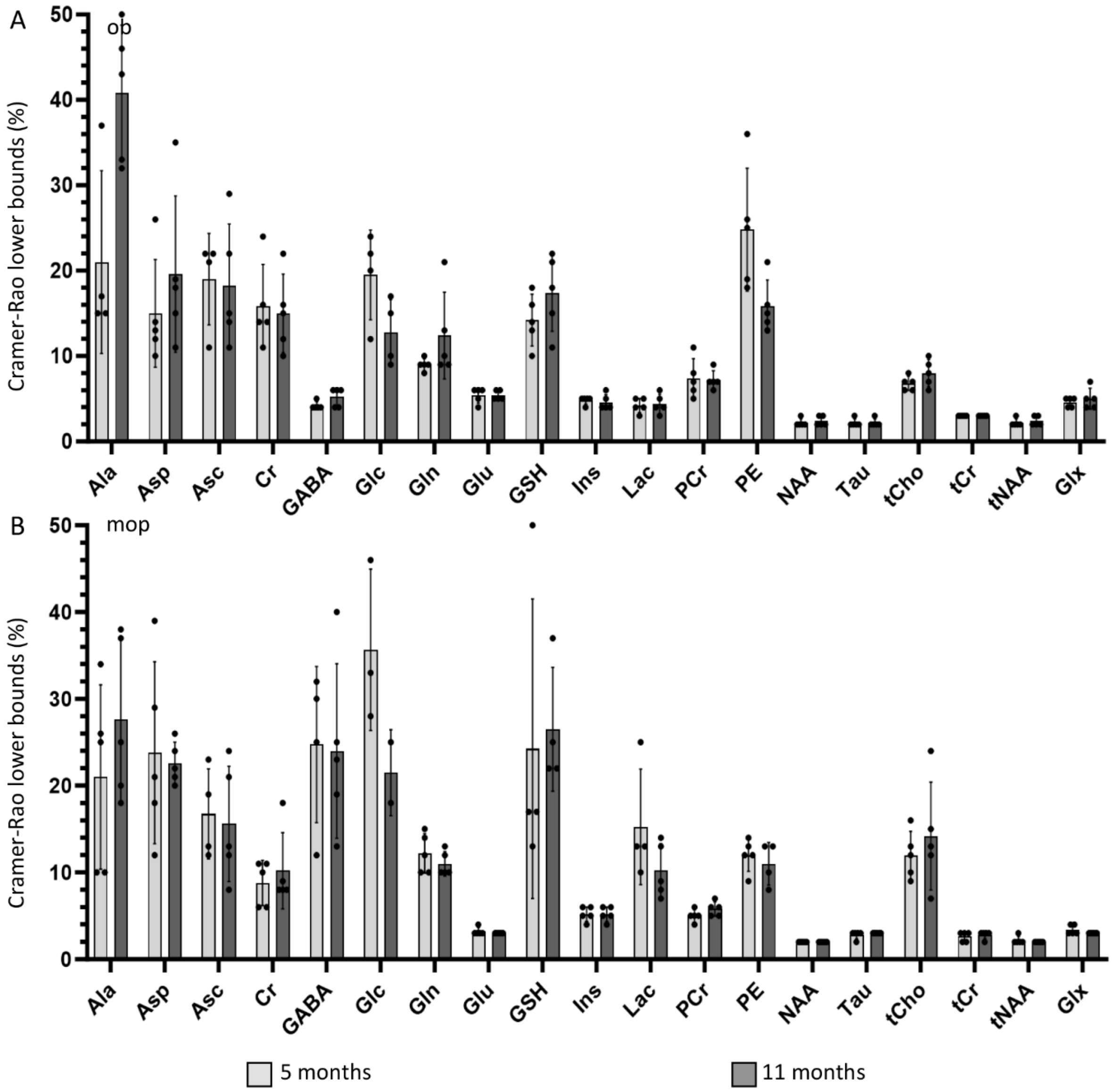

Supplementary Figure 3: Mean Cramér–Rao Lower Bound (CRLB) estimates for the metabolites quantified in Figure 3, expressed in relative units (%). Only metabolites with CRLB < 50% were considered for analysis

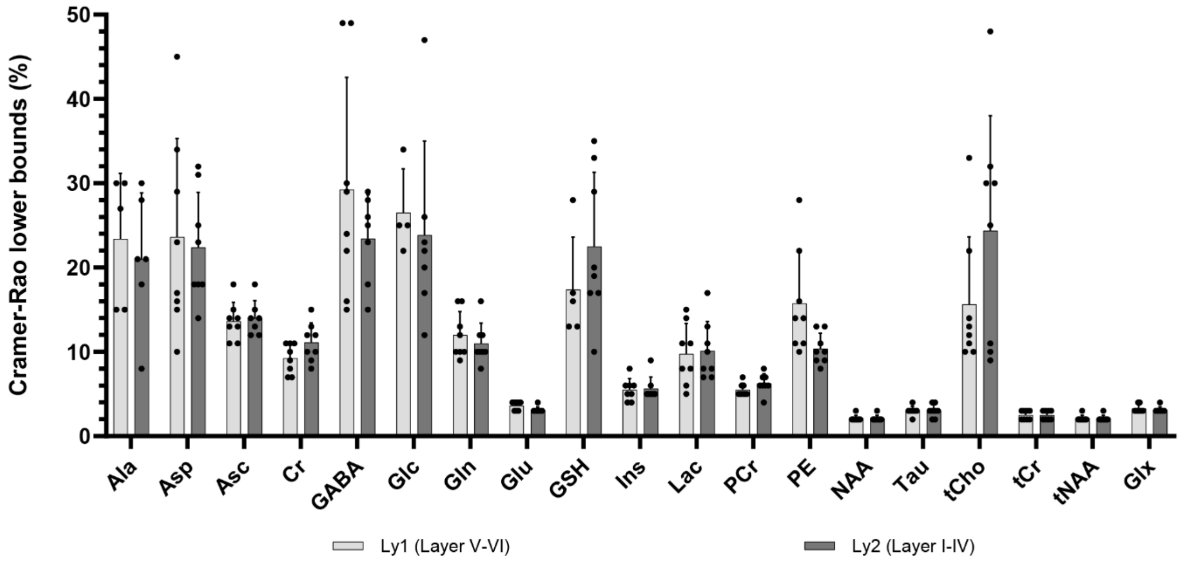

Supplement: Supplementary file 1 — Table S1. Details of the statistical analysis (two‐way ANOVA with post hoc Sidak) comparing absolute concentrations (μmol/g) of the detected analytes. Table S2: Details of the statistical analysis (two‐way ANOVA with post hoc Sidak) comparing the spectra of different cortical regions (excluding the OB). Figure S1: Mean Cramér–Rao Lower Bound (CRLB) estimates for the metabolites quantified in Figure 5, expressed in relative units (%). Only metabolites with CRLB < 50% were considered for analysis. Figure S2: Mean Cramér–Rao Lower Bound (CRLB) estimates for the metabolites quantified in Figure 6, expressed in relative units (%). Only metabolites with CRLB < 50% were considered for analysis. Figure S3: Mean Cramér–Rao Lower Bound (CRLB) estimates for the metabolites quantified in Figure 3, expressed in relative units (%). Only metabolites with CRLB < 50% were considered for analysis. [file JNC-169-0-s001.pdf]
